# Elevated LINC00894 relieves the oncogenic properties of thyroid cancer cell by sponging let-7e-5p to promote TIA-1 expression

**DOI:** 10.1007/s12672-022-00520-2

**Published:** 2022-07-01

**Authors:** Bo Chen, Deqing Liu, Runjie Chen, Libing Guo, Jianmin Ran

**Affiliations:** 1 Endocrinology Department, Guangzhou Red Cross Hospital, Medical College of Jinan University, Guangzhou, 510220 China; 2 Institute of Diseases-Oriented Nutrition Research, Guangzhou Red Cross Hospital, Medical College of Jinan University, Guangzhou, 510220 China; 3 Endocrinology Department, Guangdong Second Provincial General Hospital, Guangzhou, 510350 China; 4 Oncology Department, Guangdong Second Provincial General Hospital, 510350 Guangzhou, China

**Keywords:** Competing endogenous RNA, lncRNA, microRNA, Oncogene, Thyroid tumor

## Abstract

LINC00894 plays an important role in cancer cell proliferation and invasion in breast and kidney cancer. However, its role in thyroid cancer proliferation and metastasis remains unclear. In this study, data on LINC00894 expression in thyroid cancer tissues were obtained from GEPIA2. miRNA expression in thyroid cancer tissues was obtained from starBase 3.0 and OncomiR. Cell proliferation was evaluated using CCK-8, and Transwell chambers were used for the migration and invasion assays. LINC00894 and let-7e-5p expressions in thyroid cancer cells were measured using qRT–PCR. Meanwhile, TIA-1 expression in thyroid cancer cells was analyzed via western blotting. We found that LINC00894 expression was markedly reduced in thyroid cancer tissues and cells, and low expression of LINC00894 was associated with poor prognosis in thyroid cancer. LINC00894 overexpression inhibited the proliferation, migration, and invasion of CAL-62 and TPC-1 cells. Additionally, let-7e-5p expression was substantially enhanced in CAL-62 and TPC-1 cells. LINC00894 overexpression promoted TIA-1 expression by acting as a sponge of let-7e-5p. Finally, let-7e-5p weakened the function of LINC00894 in thyroid cancer cells via reduction in TIA-1 levels. In conclusion, our data suggest that increased LINC00894 expression reduces the oncogenic properties of thyroid cancer cells by sponging let-7e-5p to promote TIA-1 expression.

**Supplementary Information:**

The online version contains supplementary material available at 10.1007/s12672-022-00520-2.

## Introduction

In 2020, thyroid cancer was ranked ninth among all cancer types in terms of incidence (accounting for 3‒4% of all cancer cases), imposing a heavy burden on the healthcare system [1, 2]. Thyroid cancer has been categorized into several types, with papillary, follicular, and anaplastic thyroid cancers being the most common. Overall, the 5-year survival rates for patients with thyroid cancer is satisfactory (approximately 98%) [3]. However, the 5-year survival rate is poor in certain types of thyroid cancers, particularly anaplastic thyroid cancer (approximately 5%) [3]. Currently, thyroidectomy and radioactive iodine treatment are the conventional therapies for thyroid cancer [4]; however, certain types of thyroid cancer are inherently insensitive to these treatment modalities [5]. Hence, there is a need to confirm the pathogenesis of thyroid cancer to confirm novel therapeutic targets.

Long noncoding RNAs (lncRNAs) play a key regulatory role in the initiation and progression of thyroid cancer. lncRNAs act as diagnostic and prognostic biomarkers and may also function as tumor suppressors or promoters in thyroid cancer [6–8]. The long intergenic non-protein-coding RNA 894 (LINC00894) is an lncRNA that was found to be upregulated in kidney cancer and correlated with overall survival and immune infiltration [9, 10]. Additionally, LINC00894 was markedly increased in breast cancer, promoting cell proliferation and invasion [11]. Conversely, another study found that LINC00894 was strongly reduced in breast cancer cells, a phenomenon that contributed to the development of tamoxifen resistance [12]. Taken together, these studies suggest that LINC00894 plays a key regulatory role in the proliferation and drug resistance of cancer; however, the functions differ depending on the tumor type. Hence, the functional effect of LINC00894 in the occurrence and progression of thyroid cancer remains unclear.

lncRNAs play a regulatory role in tumors by functioning as miRNA sponges to competitively regulate the miRNA-targeted genes, also known as the competing endogenous RNA (ceRNA) mechanism. In thyroid cancer, AFAP1-AS1, TNRC6C-AS1, and LINC00284 regulate cancer occurrence and progression by sponging miR-204-3p, miR-513c-5p, and miR-30d-5p, respectively [13–15]. LINC00894 can sponge miR-429 to promote the progression of breast cancer [11]. To date, whether LINC00894 and miRNA sequestration affect the proliferation and metastasis in thyroid cancer remains unclear.

This study aimed to assess whether the functional effect of LINC00894 on the proliferation, migration, and invasion of thyroid cancer cells involves miRNA sponging. We first analyzed the expression of LINC00894 in the thyroid gland and its role in cancer diagnosis and prognosis using The Cancer Genome Atlas (TCGA) and Genotype-Tissue Expression (GTEx) databases. Next, we investigated the effect of LINC00894 on thyroid cancer cell proliferation, migration, and invasion. Finally, whether the function of LINC00894 in thyroid cancer is mediated via miRNA sponging was confirmed. This study may provide a novel target for the effective treatment of thyroid cancer.

## Materials and methods

### Cell culture and transfections

The thyroid follicular epithelial cell line Nthy-ori 3 − 1, anaplastic thyroid cancer line CAL-62 [include CREBBP (p.Glu1541Ter), EP300 (p.Asp1485fs), KRAS (p.Gly12Arg), NF2 (p.Glu215Ter), and TP53 (p.Ala161Asp) mutations], and papillary thyroid cancer lines TPC-1 [include CCDC6-RET fusion, CDKN2A (p.Ala68fs), STAG2 (p.Gln1089Ter), and TERT (c.1–124 C > T) mutations] and B-CPAP [include BRAF (p.Val600Glu), TERT (c.1–124 C > T), and TP53 (p.Asp259Tyr) mutations] were purchased from ZQXZ bio (Shanghai, China). CAL-62 cells were cultured in high-glucose Dulbecco’s modified Eagle medium (DMEM; ZQXZ bio), and TPC-1 and B-CPAP cells were cultured in Roswell Park Memorial Institute 1640 medium (ZQXZ bio.) supplemented with 10% fetal bovine serum at 37 °C in a humidified atmosphere containing 5% CO_2_. LINC00894 was chemically synthesized and cloned into pcDNA 3.1 to create a LINC00894 overexpression recombinant plasmid (LINC00894-OE group). Empty pcDNA 3.1 vector was used as a negative control (EP-OE group). Let-7e-5p mimic (let-7e-5p mimic group) and negative control mimic (NC mimic group) were purchased from Ribobio (Guangzhou, China). Next, NC-OE, LINC00894-OE, LINC00894-OE and NC mimics as well as LINC00894-OE and let-7e-5p mimics were separately transfected into CAL-62 and TPC-1 cells using Lipofectamine 3000 reagent (Invitrogen, Carlsbad, CA, USA). At 24 h post transfection, the transfected cells were used for further experiments.

### Cell proliferation, migration, and invasion assay

The proliferation of CAL-62 and TPC-1 cells was measured using Cell Counting Kit-8 (CCK-8; Dojindo, Kumamoto, Japan) at 450 nm [16] 24, 48, and 72 h post transfection. Transwell chambers (8 μm pore-size; Corning Costar, Cambridge, MA) were used for the migration and invasion assays [16]. For the cell invasion assay, the chambers were first coated with 0.1 mL of Matrigel for 1 h at 37 °C. For the cell migration assay, the chambers did not need to be coated. The transfected cells were seeded in the upper chamber of the well. After 24 h of incubation, the migratory and invading cells on the lower surface of the insert membrane were stained with 0.1% crystal violet. The stained cells were then photographed in three randomly chosen visual fields and counted. Three replicates were performed for each group in the assay.

### Quantitative reverse transcription polymerase chain reaction (qRT–PCR)

Total RNA was isolated from TPC-1 and CAL-62 cells using TRIzol reagent (Invitrogen). RNA was reverse transcribed into cDNA using PrimeScript RT reagent kit (TAKARA, Japan), and qRT–PCR was performed using AceQ qPCR SYBR Green Master Mix (Vazyme) according to the manufacturer’s instructions. mRNA expression levels were quantified using the 2^−ΔΔCt^ method [17]. β-actin and U6 served as internal controls for the normalization of LINC00894 and let-7e-5p, respectively. The following forward and reverse primer sequences were used: LINC00894: 5′-CCAAATCTGACACACCATAGC-3′ and 5′-GAACACAGCATGCAGGTAAT-3′; β-actin: 5′-TGGATCAGCAAGCAGGAGTA-3′ and 5′-TCGGCCACATTGTGAACTTT-3′; let-7e-5p: 5′- ACACTCCAGCTGGGTGAGGTAGGAGGTTGTAT-3′ and 5′-CTCAACTGGTGTCGTGGA-3′; U6: 5′- CTCGCTTCGGCAGCACA-3′ and 5′-AACGCTTCACGAATTTGCGT-3′.

### Luciferase reporter and AGO2-RNA immunoprecipitation (RIP) assays

The lncLocator web tool was used to predict the subcellular localization of LINC00894. starBase 3.0 and RNAInter databases were used to identify potential miRNA-LINC00894 interactions [18, 19], whereas TargetScan, starBase 3.0, and RNAInter databases were used to identify potential let-7e-5p-mRNA interactions [18–20]. After the analysis, wild-type LINC00894 and the 3′-UTR of cytotoxic granule-associated RNA-binding protein (TIA-1; WT-LINC00894 and WT-TIA-1, respectively) and mutated LINC00894 and TIA-1 (Mut-LINC00894 and Mut-TIA-1, respectively) were cloned into psi-CHECK2 vectors and cotransfected into 293T cells using Lipofectamine 3000 (Invitrogen, Carlsbad, CA, USA) along with let-7e-5p mimic or NC mimic. Finally, the ratio of *Renilla*-to-firefly luciferase activity was evaluated using the Dual-Luciferase Reporter Assay System (Promega) 24 h after transfection. AGO2-RIP assay was performed using EZ-Magna RIP RNA-Binding Protein Immunoprecipitation Kit (Merck Millipore, Bedford, MA, USA) following the manufacturer’s instructions. In addition, LINC00894 and let-7e-5p expression was measured by qRT–PCR.

### Western blotting

Proteins were extracted using radioimmunoprecipitation assay buffer (Beyotime, Shanghai, China) and separated by 10% sodium dodecyl sulfate-polyacrylamide gel electrophoresis. The separated proteins were transferred onto polyvinylidene fluoride membranes (Millipore, MA, USA), which were subsequently incubated with the following primary antibodies at 37 °C for 2 h: anti-TIA-1 (Abcam, ab263945; 1:1000) and anti-GAPDH (Abcam, ab181602; 1:10,000). The membranes were then incubated with appropriate horseradish peroxidase‒conjugated secondary antibodies (Abcam, ab205718, 1:50,000) at 25 ℃ for 1 h. Target proteins were visualized using a chemiluminescence substrate (Keygentec, Jiangsu, China), and the chemiluminescence signal was detected via exposure to X-ray film.

### Statistical analysis

LINC00894 and TIA-1 expressions were obtained from the Gene Expression Profiling Interactive Analysis 2 (GEPIA2) database, a web-based tool based on TCGA and GTEx databases [21]. miRNA expression was obtained from starBase 3.0 and OncomiR databases [18, 22]. Survival analysis was conducted using Kaplan–Meier plotter database [23]. The diagnostic value of LINC00894 in thyroid cancer was assessed using receiver operating characteristic (ROC) analysis. Normally distributed data (three replicates) in this study are presented as the mean ± standard deviation. Multiple groups were compared using one-way analysis of variance followed by Tukey’s post hoc test. Significance was set at *p* < 0.05.

## Results

### LINC00894 expression is reduced in thyroid cancer

**Fig. 1:**
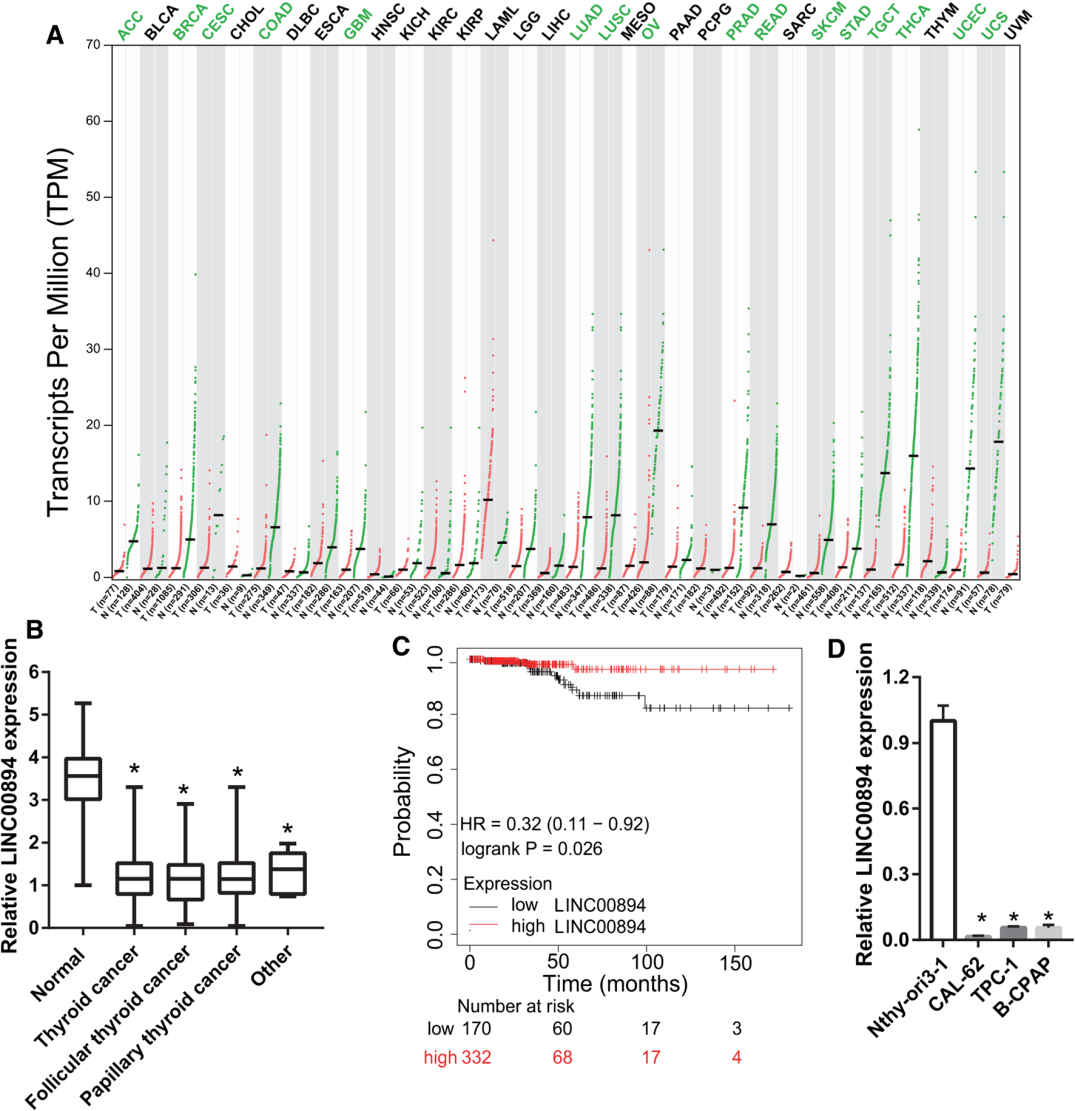
LINC00894 expression is reduced in thyroid cancer. **A** LINC00894 expression in cancer tissues was analyzed using GEPIA2. The green color indicates low LINC00894 expression in cancer. T: tumor tissue; N: normal tissue. **B** LINC00894 expression in thyroid cancer tissues (n = 512) include follicular thyroid cancer (n = 106), papillary thyroid cancer (n = 397), and other thyroid cancer (n = 9) and normal tissues (n = 712) was analyzed. **C** The prognostic value of LINC00894 was determined using the Kaplan–Meier plotter. **D** LINC00894 expression in thyroid cancer cell lines (CAL-62, TPC-1, and B-CPAP) and in a nontumorigenic thyroid cell line (Nthy-ori 3-1) was analyzed using quantitative reverse transcription polymerase chain reaction (qRT–PCR). *p < 0.05

To study LINC00894 expression and its clinical function in thyroid carcinoma, we analyzed its expression profiles in various cancer tissues using GEPIA2. The results revealed that LINC00894 expression was significantly downregulated in many types of cancer, including thyroid cancer (Fig. 1A and B). LINC00894 expression was significantly downregulated in follicular thyroid cancer and papillary thyroid cancer tissues compared with normal tissues (Fig. 1B). LINC00894 expression had no significantly changed between follicular thyroid cancer, papillary thyroid cancer, and other thyroid cancer (Fig. 1B). In addition, Kaplan–Meier survival analysis showed that low expression of LINC00894 was associated with poor survival in patients with thyroid cancer (Fig. 1C). Furthermore, LINC00894 expression in thyroid cancer cell lines (CAL-62, TPC-1, and B-CPAP) was significantly lower than that in normal thyroid cell lines (Nthy-ori-3-1; Fig. 1D).

### LINC00894 overexpression inhibits the biological behavior of thyroid cancer cells

**Fig. 2:**
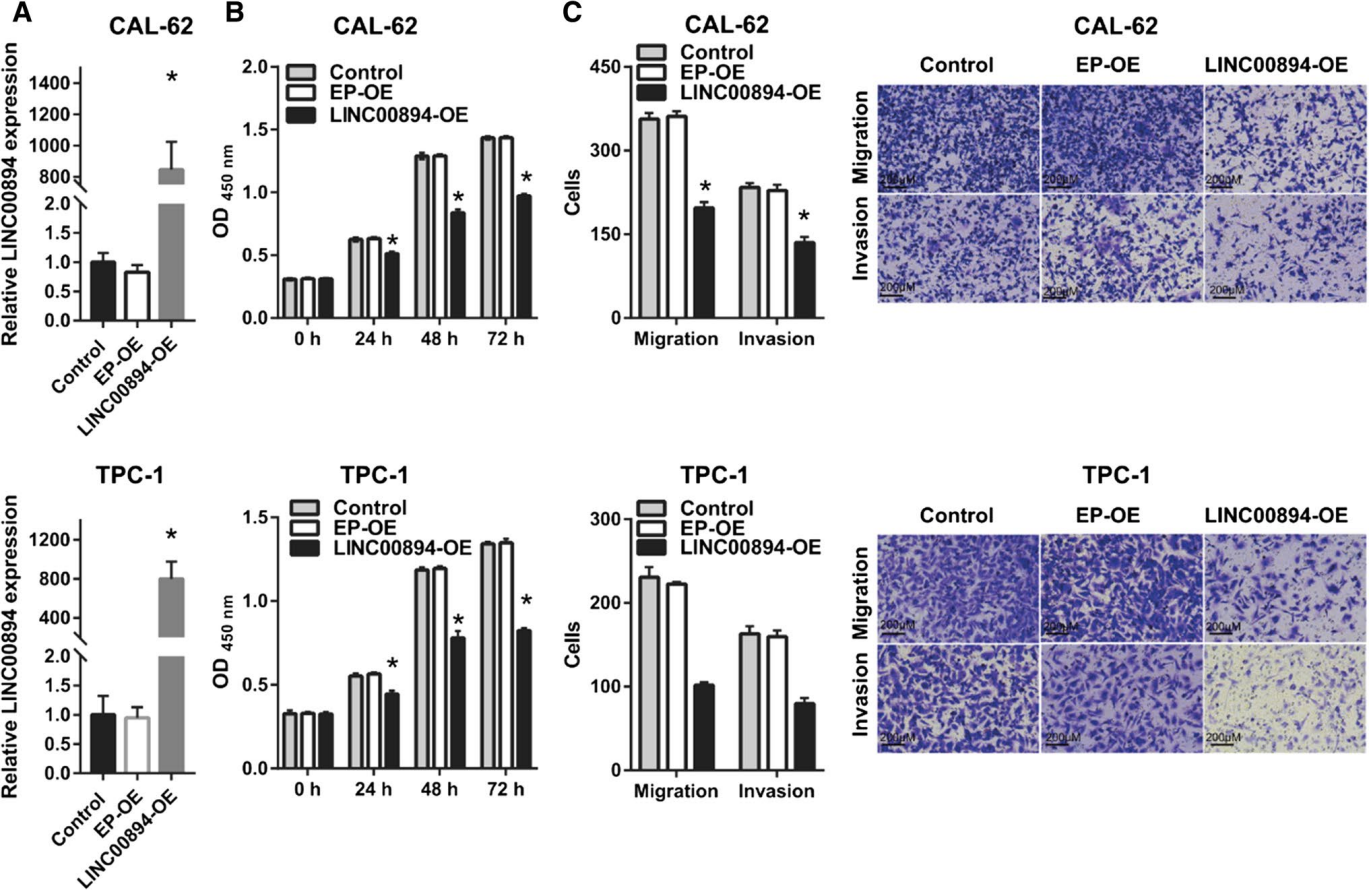
LINC00894 overexpression reduced the biological behavior of CAL-62 and TPC-1 cells. **A** LINC00894 expression was determined by qRT–PCR in thyroid cancer 24 h after transfection. **B** and **C** The proliferation (**B**) and migration and invasion (**C**, magnification ×100) of CAL-62 and TPC-1 cells were evaluated using the CCK-8 Kit and Transwell chambers, respectively, 24 h after transfection. *p < 0.05

To elucidate effect of LINC00894 expression on thyroid cancer cells, LINC00894-OE was transfected into CAL-62 and TPC-1 cells. Next, LINC00894 overexpression in CAL-62 and TPC-1 cells was verified, and it was found that LINC00894 expression was significantly increased in the LINC00894-OE group (Fig. 2A). Compared with those in the EP-OE group, the proliferation, migration, and invasion of CAL-62 and TPC-1 cells was significantly inhibited in the LINC00894-OE group (Fig. 2B, C).

### LINC00894 regulates TIA-1 expression by acting as a sponge of let-7e-5p

**Fig. 3:**
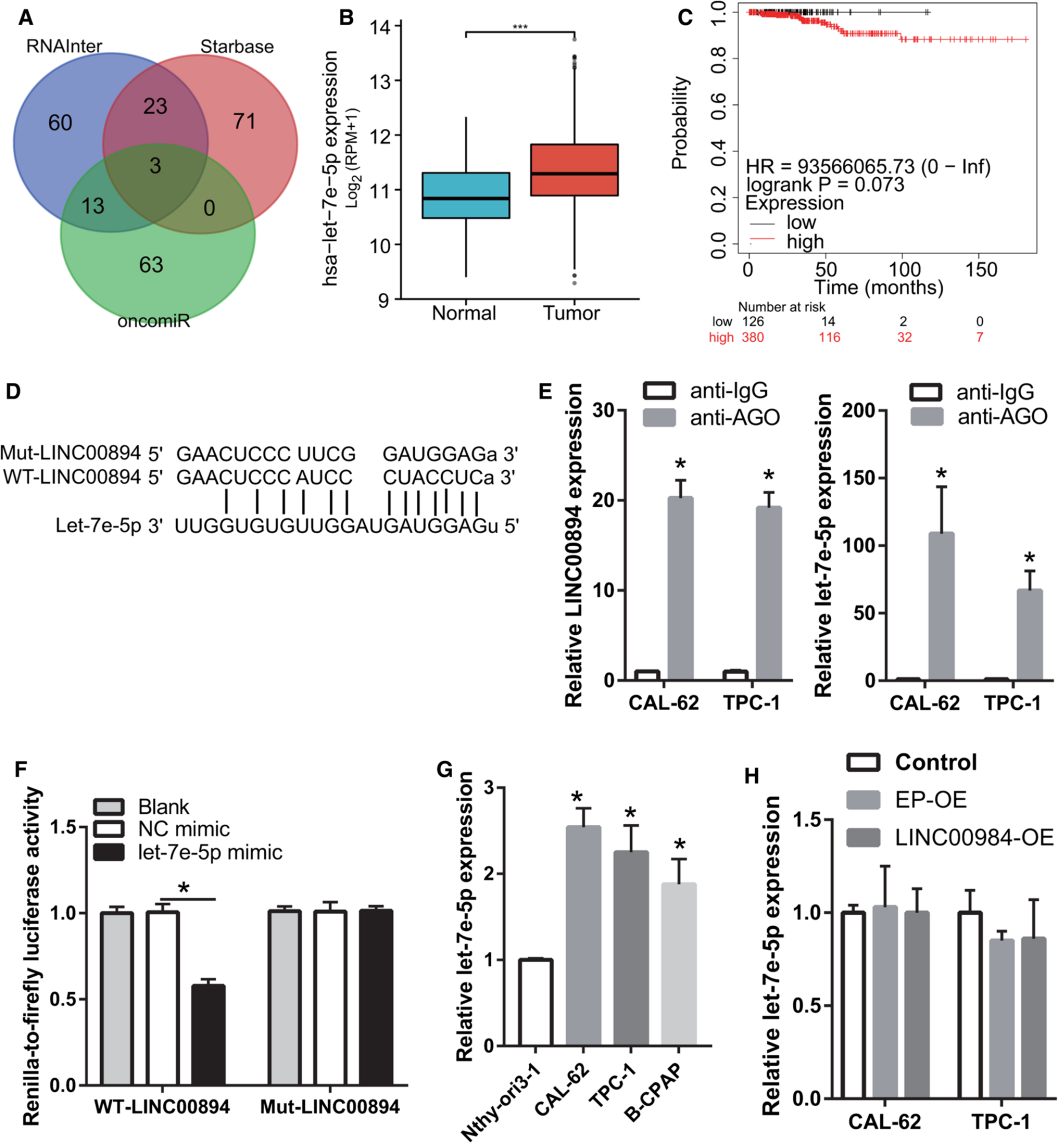
LINC00894 acts as a sponge of let-7e-5p. **A** Three potential miRNAs bound by LINC00894 were identified using oncomiR, starBase 3.0, and RNAInter. **B** let-7e-5p expression was analyzed using StarBase 3.0. **C** The prognostic value of let-7e-5p in thyroid cancer was analyzed using Kaplan–Meier plotter. **D** The binding and mutational sites of LINC00894 that bound with let-7e-5p are shown. **E** Ago-RIP analysis confirmed the interaction between let-7e-5p and LINC00894. **F** Luciferase reporter gene assay confirmed targeted binding between let-7e-5p and LINC00894. **G** Let-7e-5p expression in thyroid cancer cell lines (CAL-62, TPC-1, and B-CPAP) and in a nontumorigenic thyroid cell line (Nthy-ori 3-1) was analyzed by qRT–PCR. **H** let-7e-5p expression in CAL-62 and TPC-1 cells was analyzed by qRT–PCR 24 h post transfection. *p < 0.05

LINC00894 expression was primarily localized in the cytoplasm as revealed by the analysis using the lncLocator website, suggesting that LINC00894 primarily functions by adsorbing miRNA. StarBase 3.0, RNAInter, and oncomiR analysis revealed three potential miRNAs (miR-424-5p, miR-15a-5p, and let-7e-5p) sponged by LINC00894 (Fig. 3A). As LINC00894 was found to be expressed at a low level, miRNAs that were expressed at high levels may be potential target genes of LINC00894 in thyroid cancer. Among the three miRNAs, let-7e-5p was significantly upregulated in thyroid cancer. In addition, a high level of let-7e-5p was correlated with poor prognosis in thyroid cancer according to starBase 3.0 and Kaplan–Meier survival analyses (Fig. 3B, C), suggesting that let-7e-5p is the main miRNA adsorbed by LINC00894 in thyroid cancer. Therefore, let-7e-5p was chosen as the potential target gene of LINC00894. The binding and mutational sites of LINC00894 which bound with let-7e-5p are shown in Fig. 3D. Ago-RIP analysis showed that LINC00894 and let-7e-5p expression was more abundant in the anti-AGO2 antibodies group than in the anti-IgG group, suggesting that LINC00894 binds to let-7e-5p via AGO2 (Fig. 3E). Compared to that in the WT-LINC00894 + NC mimic group, the *Renilla*-to-firefly luciferase activity in the WT-LINC00894 + let-7e-5p mimic group was reduced but was not significantly different between the Mut-LINC00894 + NC and Mut-LINC00894 + let-7e-5p mimic groups, indicating that LINC00894 can bind to let-7e-5p (Fig. 3F). Moreover, let-7e-5p was highly expressed in thyroid cancer cells, which is consistent with its expression in thyroid cancer tissues (Fig. 3G). Additionally, it was demonstrated that LINC00894 overexpression did not affect let-7e-5p expression (Fig. 3H). These results show that LINC00894 only adsorbs let-7e-5p without affecting its expression.

**Fig. 4:**
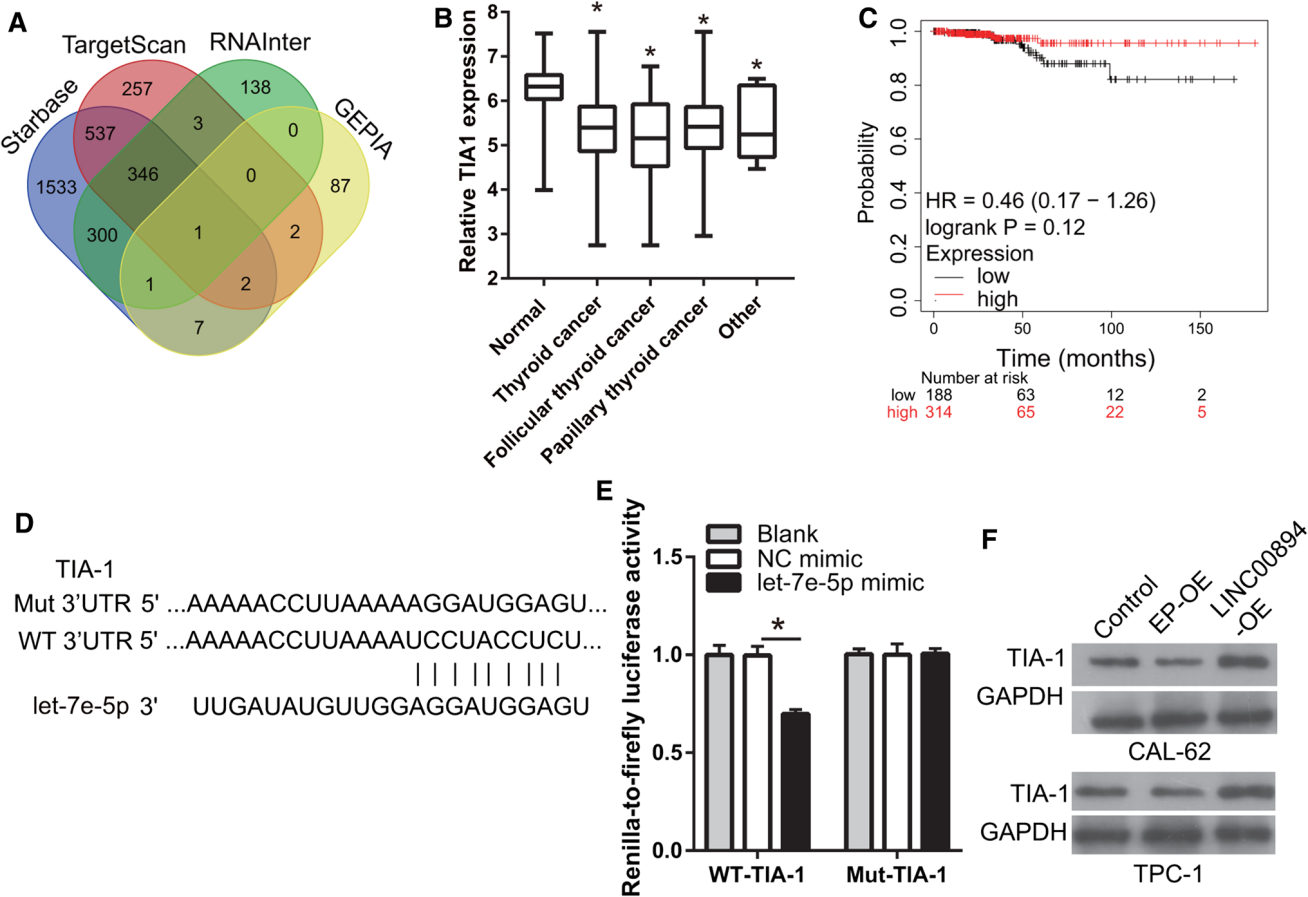
**T**IA-1 is the gene target of let-7e-5p. **A** Potential target genes bound by let-7e-5p were identified using starBase 3.0, TargetScan 8.0, and GEPIA2. **B** TIA-1 expression in thyroid cancer tissues (n = 512) include follicular thyroid cancer (n = 106), papillary thyroid cancer (n = 397), and other thyroid cancer (n = 9) and normal tissues (n = 712) was investigated. **C** The prognostic value of TIA-1 in thyroid cancer was analyzed using Kaplan–Meier plotter. **D** The binding sites between let-7e-5p and the 3′-UTR of TIA-1 are shown. **E** The binding between let-7e-5p and the 3′-UTR of TIA-1 was determined using a luciferase assay. **F** TIA-1 expression in CAL-62 and TPC-1 cells was analyzed by western blotting 24 h after transfection. *p < 0.05

Additional potential target genes regulated by let-7e-5p were investigated using starBase 3.0, TargetScan 8.0, and RNAInter, and those which had a significantly positive correlation with LINC00894 expression in thyroid cancer were analyzed using GEPIA2. TIA-1 was identified at the intersection of the four groups (Fig. 4A). TIA-1 was significantly downregulated in thyroid cancer include follicular thyroid cancer and papillary thyroid cancer and this low TIA1expression was related to poor prognosis according to GEPIA2 and Kaplan–Meier survival analyses (Fig. 4B, C). TIA-1 expression had no significantly changed between follicular thyroid cancer, papillary thyroid cancer, and other thyroid cancer (Fig. 4B). These results indicate that TIA-1 and LINC00894 expressions are significantly related and that TIA-1 is a potential target gene for let-7e-5p in thyroid cancer. The binding sites of let-7e-5p and TIA-1 are shown in Fig. 4D. The luciferase assay showed that luciferase activity was significantly decreased in the WT-TIA-1 + let-7e-5p mimic group compared with that in the WT-TIA-1 + NC mimic group but was not significantly different between the Mut-TIA-1 + NC mimic and Mut-TIA-1 + let-7e-5p mimic groups, indicating that the 3′-UTR of TIA-1 can bind to let-7e-5p (Fig. 4E). Moreover, overexpression of LINC00894 promoted TIA-1 expression (Fig. 4F). These results indicate that LINC00894 and TIA-1 3-UTR have a ceRNA regulatory relationship by binding the same let-7e-5p and that LINC00894 increases TIA-1 protein expression by sponging let-7e-5p in thyroid cancer cells.

### Let-7e-5p weakens LINC00894 function by reducing TIA-1 expression

**Fig. 5:**
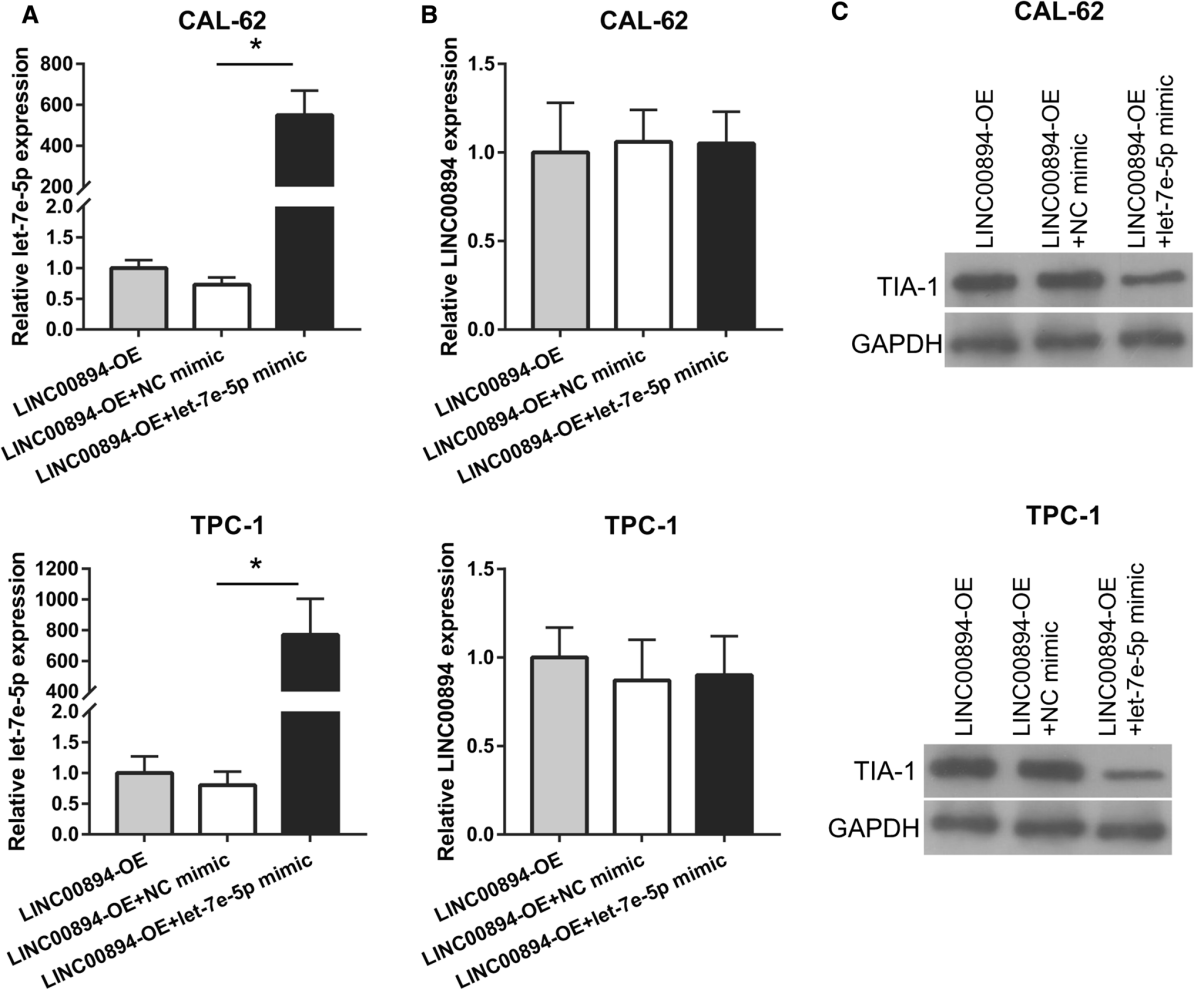
let-7e-5p overexpression results in the reduction of TIA-1 levels but does not affect LINC00894 expression. **A**, **B** let-7e-5p (**A**) and LINC00894 (**B**) expression was analyzed by qRT–PCR in thyroid cancer cells 24 h after cotransfection. **C** TIA-1 levels in CAL-62 and TPC-1 cells were analyzed by western blotting 24 h after cotransfection. *p < 0.05

**Fig. 6:**
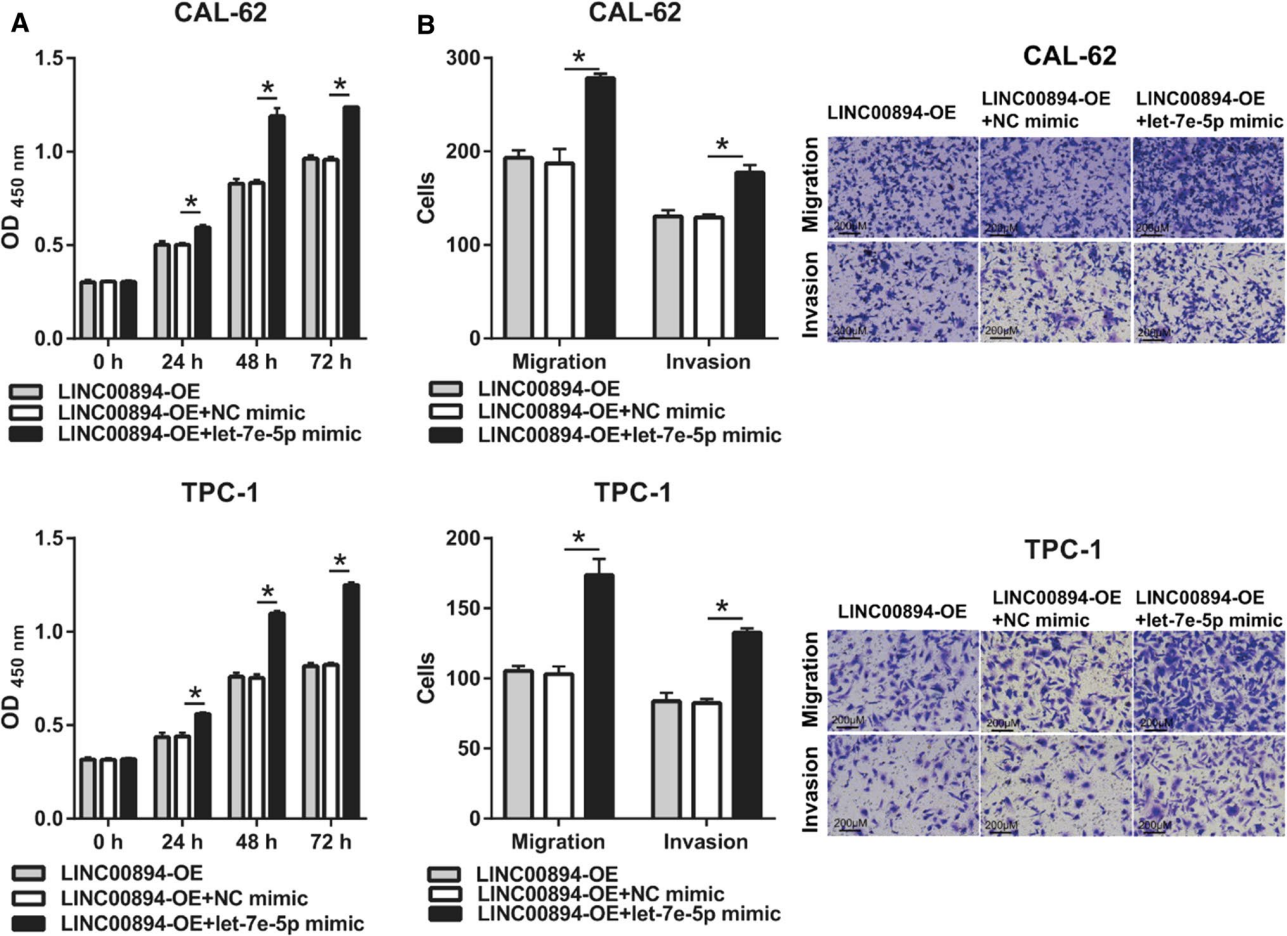
let-7e-5p overexpression promotes the biological behavior of LINC00894-OE–transfected CAL-62 and TPC-1 cells. **A** Cell proliferation was quantified using the CCK-8 kit 24 h after cotransfection. **B** Migration and invasion were determined using Transwell chambers 24 h post transfection (magnification × 100). *p < 0.05

To further confirm that let-7e-5p is the downstream functional gene of LINC00894, we cotransfected LINC00894-OE and let-7e-5p mimic and analyzed whether let-7e-5p could weaken the function of LINC00894. Compared with that in the LINC00894-OE + NC mimic group, the expression of let-7e-5p and TIA-1 was significantly increased, whereas LINC00894 expression did not significantly change let-7e-5p expression (Fig. 5A–C). Moreover, our results demonstrated that the proliferation, migration, and invasion of CAL-62 and TPC-1 cells was significantly reduced in the LINC00894-OV + let-7e-5p group compared to that in the LINC00894-OE + NC mimic group (Fig. 6A and B).

## Discussion

LncRNAs serve as potential biomarkers for diagnosis and prognosis and as targets for treatment in thyroid cancer [6, 24]. In this study, LINC00894 was significantly downregulated in different cancers, including thyroid cancer, indicating its potential roles as a putative tumor suppressor. Additionally, LINC00894 was found to accurately predict thyroid cancer prognosis. Finally, LINC00894 inhibited thyroid cancer cell proliferation, migration, invasion by acting as a sponge of let-7e-5p to promote TIA-1 expression. Hence, we discovered a novel mechanism regulating the proliferation, migration, and invasion of thyroid cancer cells.

Previous studies have shown that LINC00894 plays various roles in different tumors. In breast and renal cancers, LINC00894 was upregulated, a phenomenon that was correlated with overall survival and promoted cell proliferation and invasion, suggesting that it could function as an oncogene [9–11]. In contrast, LINC00894 contributed to reversing tamoxifen resistance in breast cancer [12]. In this study, LINC00894 was significantly downregulated in thyroid cancer tissues and cells, and its overexpression resulted in inhibition of cell proliferation, migration, and invasion. This study reveals a novel function of LINC00894 as an antioncogene to inhibit tumor cell proliferation, migration, and invasion in thyroid cancer.

According to the ceRNA hypothesis, lncRNAs compete with 3′-UTR of mRNAs by binding with miRNAs, thereby regulating the translation of mRNA into protein and playing a key role in tumorigenesis. In this study, LINC00894 bound with let-7e-5p. In addition, we found let-7e-5p overexpression did not affect LINC00894 expression and vice versa, demonstrating that LINC00894 and let-7e-5p only bind to each other but do not affect their respective expression. Previous studies have demonstrated that let-7e-5p was significantly downregulated and let-7e-5p inhibited tumorigenesis and aggressive behavior in head and neck squamous cell carcinoma and non-small cell lung cancer [25, 26]. Conversely, let-7e-5p was significantly upregulated in young patients with oral cavity cancer, colorectal cancer, and rectal carcinoma with liver metastases and promoted the tumorigenesis and aggressive behavior of cancer cells [27–29]. Similarly, let-7e-5p was overexpressed in thyroid cancer cells, and a high level of let-7e-5p was correlated with poor prognosis. Additionally, let-7e-5p overexpression weakened the function but did not affect the expression of LINC00894. This study demonstrates that let-7e-5p promotes tumorigenesis and aggressive behavior in thyroid cancer and LINC00894 inhibits the progression of thyroid cancer via adsorption of let-7e-5p. TIA-1 is an RNA-binding protein that was found to be a relatively new tumor suppressor in patients with lung squamous cell carcinoma [30]. Loss of TIA-1 expression promoted the tumorigenesis and aggressive behavior of pancreatic cancer cells [31]. TIA-1 levels were reduced by miR-19a and miR-487a in breast and gastric cancers, respectively, thereby promoting cell migration and invasion [32, 33]. This study demonstrated that TIA-1 protein expression increased upon LINC00894 overexpression but was inhibited by let-7e-5p overexpression. Additionally, let-7e-5p can bind to the 3′-UTR of TIA-1. These results suggest that LINC00984 and the 3′-UTR of TIA-1 have a ceRNA regulatory relationship by binding let-7e-5p and LINC00894 increases the level of TIA-1 by sponging let-7e-5p.

Despite these findings, this study has limitations. First, we did not collect any clinical specimens to further verify LINC00894 expression in thyroid cancer tissues and to validate whether LINC00894 can be used as a diagnostic and prognostic marker. Second, we did not perform in vivo experiments to verify the effect of the LINC00894/let-7e-5p/TIA-1 pathway on the growth and metastasis of thyroid cancer.

## Conclusion

**Fig. 7:**
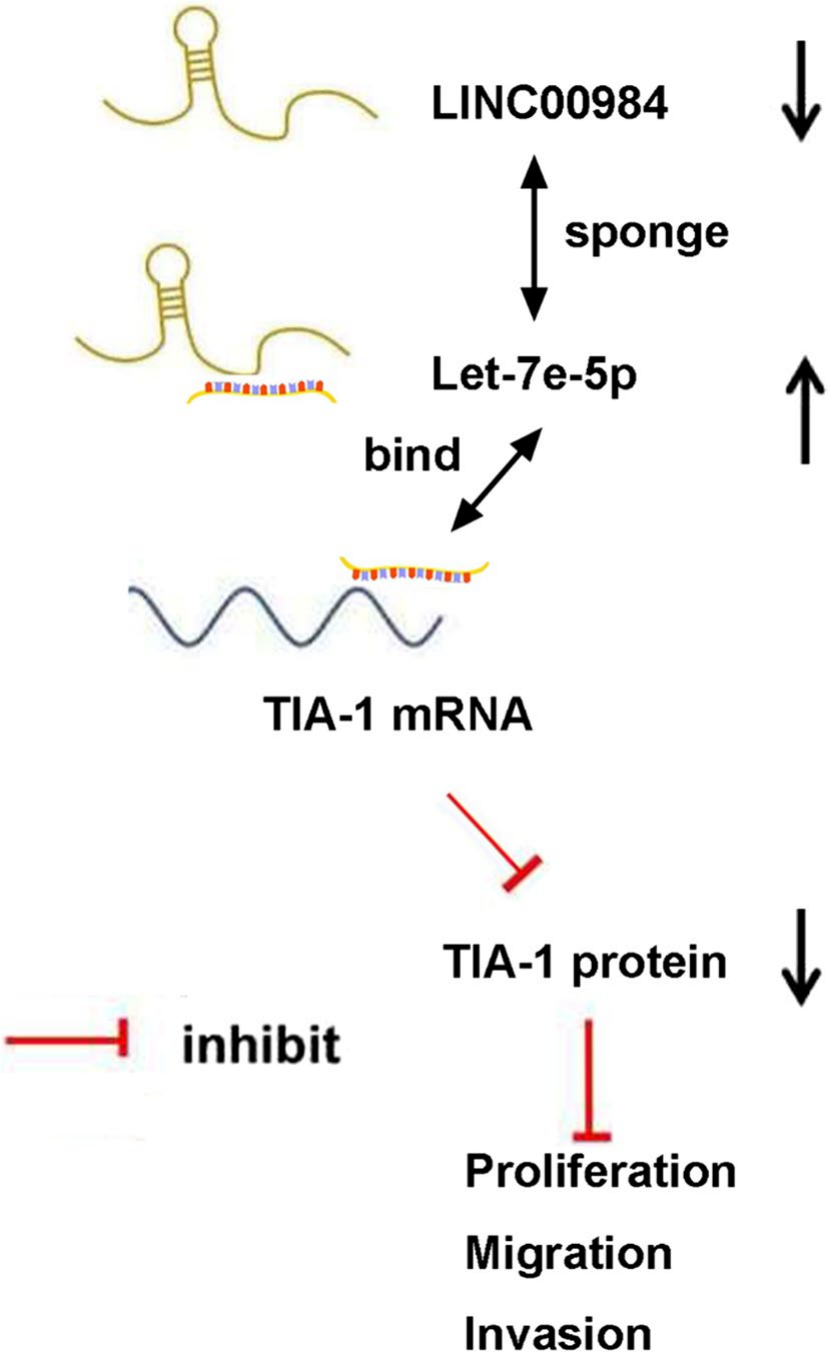
Regulatory mechanism: LINC00894 was downregulated in thyroid cancer tissues and inhibited cancer cell proliferation, migration, and invasion by acting as a sponge of let-7e-5p to regulate TIA-1 expression. LINC00984 and the 3′-UTR of TIA-1 have a ceRNA regulatory relationship by binding the same miRNA, let-7e-5p. In cell homeostasis, the let-7e-5p bound by LINC00984 and those bound by the 3′-UTR of TIA-1 are balanced. In thyroid cancer. Upon reduced LINC00984 expression, low let-7e-5p bind to LINC00894, resulting in more let-7e-5p bound to TIA-1 3′-UTR and decreased TIA-1 expression; Upon increased LINC00984 expression, more let-7e-5p bind to LINC00894, resulting in fewer let-7e-5p bound to TIA-1 3’-UTR and increased TIA-1 expression

LINC00894 was downregulated in thyroid cancer tissues and inhibited the cancer cell proliferation, migration, and invasion by acting as a sponge of let-7e-5p to regulate TIA-1 protein levels (Fig. 7). These data suggest that LINC00894 acts as an antioncogene and may be a novel therapeutic target for thyroid cancer.

## Supplementary Information

Below is the link to the electronic supplementary material.

Supplementary material 1 (PDF 377.5 kb)

## Data Availability

All data during the study appear in the submitted article.

## Abbreviations

lncRNA: Long noncoding RNA
LINC00894: Long intergenic non-protein-coding RNA 894
TCGA: The Cancer Genome Atlas
GTEx: Genotype-Tissue Expression
NC: Negative control
TIA-1: Cytotoxic granule-associated RNA-binding protein
GEPIA2: Gene Expression Profiling Interactive Analysis 2
ROC: Receiver operating characteristic
