# Supplementary figures and images for: Elevated LINC00894 relieves the oncogenic properties of thyroid cancer cell by sponging let-7e-5p to promote TIA-1 expression
Source: Discov Oncol. 2022 Jul 1;13:56. doi: 10.1007/s12672-022-00520-2 (PMC9249958; doi:10.1007/s12672-022-00520-2)

**Figure 4F**

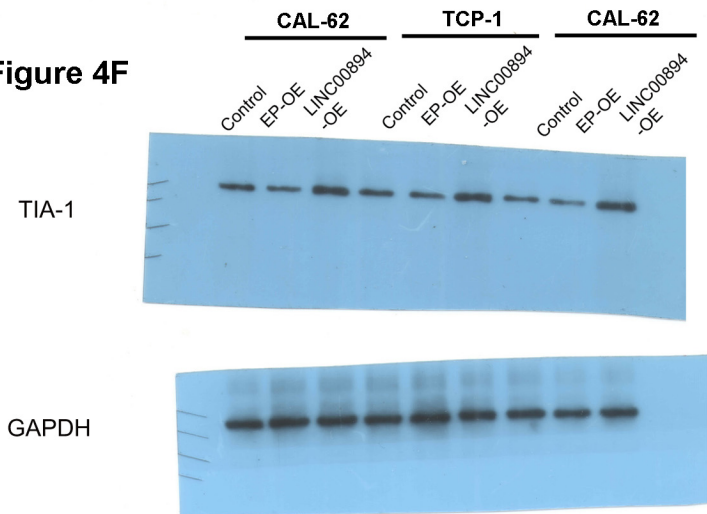

**Figure 5C**

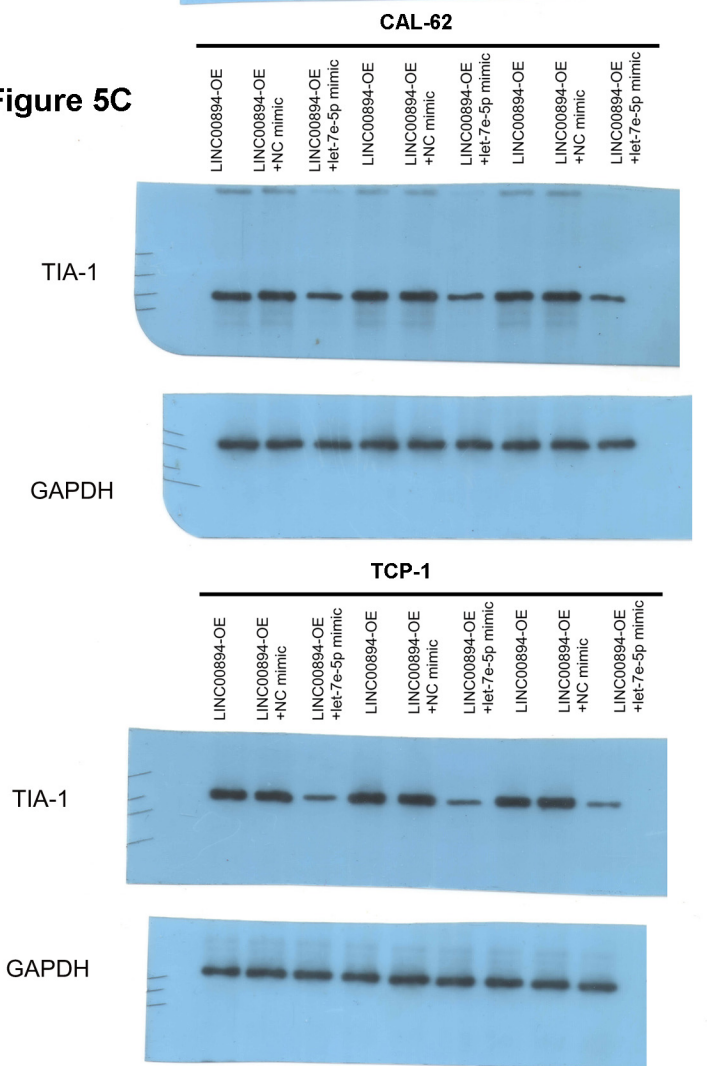

Supplement: Supplementary file 1 — Supplementary material 1 (PDF 377.5 kb) [file 12672_2022_520_MOESM1_ESM.pdf]
